# Plant catalases as NO and H_2_S targets

**DOI:** 10.1016/j.redox.2020.101525

**Published:** 2020-05-25

**Authors:** José M. Palma, Rosa M. Mateos, Javier López-Jaramillo, Marta Rodríguez-Ruiz, Salvador González-Gordo, Alfonso M. Lechuga-Sancho, Francisco J. Corpas

**Affiliations:** a Group of Antioxidants, Free Radicals and Nitric Oxide in Biotechnology, Food and Agriculture, Dept. Biochemistry, Cell and Molecular Biology of Plants, Estación Experimental del Zaidín, CSIC, Granada, Spain; b Imflammation, Nutrition, Metabolism and Oxidative Stress Study Group (INMOX), Biomedical Research and Innovation Institute of Cádiz (INiBICA), Research Unit, Puerta del Mar University Hospital, Cádiz, Spain; c Area of Biochemistry and Molecular Biology, Department of Biomedicine, Biotechnology and Public Health, University of Cádiz, Cádiz, Spain; d Instituto de Biotecnología, Universidad de Granada, Spain; e Laboratório de Fisiologia do Desenvolvimiento Vegetal; Instituto de Biociências-Universidad de São Paulo; Cidade Universitária-São Paulo-SP, Brazil; f Department of Child and Mother Health and Radiology, Medical School, University of Cádiz, Cádiz, Spain

**Keywords:** Docking, Nitration, S-nitrosation, Persulfidation, Post-translational modifications, Signaling

## Abstract

Catalase is a powerful antioxidant metalloenzyme located in peroxisomes which also plays a central role in signaling processes under physiological and adverse situations. Whereas animals contain a single catalase gene, in plants this enzyme is encoded by a multigene family providing multiple isoenzymes whose number varies depending on the species, and their expression is regulated according to their tissue/organ distribution and the environmental conditions. This enzyme can be modulated by reactive oxygen and nitrogen species (ROS/RNS) as well as by hydrogen sulfide (H_2_S). Catalase is the major protein undergoing Tyr-nitration [post-translational modification (PTM) promoted by RNS] during fruit ripening, but the enzyme from diverse sources is also susceptible to undergo other activity-modifying PTMs. Data on *S*-nitrosation and persulfidation of catalase from different plant origins are given and compared here with results from obese children where S-nitrosation of catalase occurs. The cysteine residues prone to be S-nitrosated in catalase from plants and from bovine liver have been identified. These evidences assign to peroxisomes a crucial statement in the signaling crossroads among relevant molecules (NO and H_2_S), since catalase is allocated in these organelles. This review depicts a scenario where the regulation of catalase through PTMs, especially S-nitrosation and persulfidation, is highlighted.

## Highlights

• This work reviews molecular features of catalase from plant origin.
• Catalase activity is regulated in plants by diverse post-translational modifications.
• Plant catalases are targets of nitration, S-nitrosation, and persulfidation events.
• Human catalase was also proved to be S-nitrosated in obese children.
• Nitrosated cysteines from plant catalases were identified.

## Graphical abstract

**Figure:**
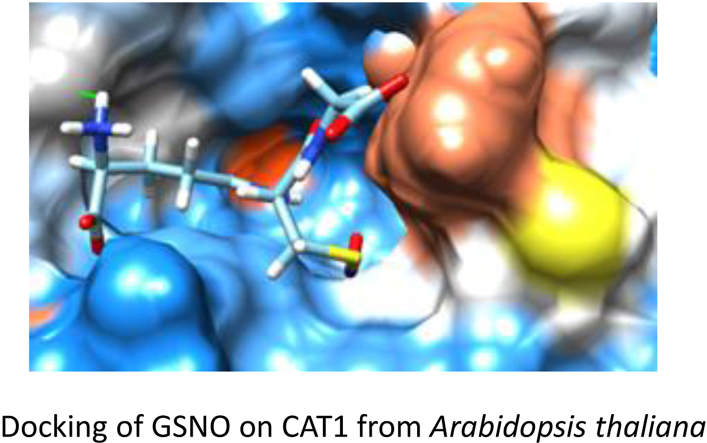

## 1 Introduction

Catalase (CAT; EC 1.11.1.6) is basically an iron-containing homotetrameric protein which catalyzes the dismutation of H_2_O_2_ into H_2_O and O_2_ and plays a central role in the antioxidative metabolic network of prokaryotic and eukaryotic cells [21,24,49,53,61,82]. As a matter of fact, by virtue of its kinetic properties, catalase maintains the H_2_O_2_ levels under certain limits and, accordingly, is considered as one of the main antioxidant enzymes with a key role under developmental and stress conditions [4,43,44,85,93,118]. However, this is not the only enzymatic system which scavenges H_2_O_2_ in living beings. In animal cells, besides CAT, either selenium-dependent or selenium-independent glutathione peroxidases (GPX; EC 1.11.1.9) also participate to bring this ROS down to physiological concentrations [53,106]. The need to maintain these Se-GPXs active explains the nutritional dependence of Se in our diet. Some peroxiredoxins (Prxs), small proteins with peroxidase activity, contribute as well to detoxify intracellular H_2_O_2_ excess [60,69,106]. Even, it has been reported that Prx1 from vertebrates, such as the puffer fish (*Tetraodon nigroviridis*) and humans, shows Fe-dependent catalase-like activity, but independent of Cys residues or reducing agents [119,120].

In plants, other enzymes, mainly displaying peroxidase activity, help catalase to modulate the hydrogen peroxide concentration within cells [93,100,116]. In most cases, catalase, due to its high K_M_, scavenges the major part of H_2_O_2_ which is driven to peroxisomes, whereas enzymes such as ascorbate peroxidase (APX, EC. 1.11.1.11), which has higher affinity for H_2_O_2_ (lower K_M_) and is located in diverse subcellular compartments (mainly in plastids, mitochondria, peroxisomes and apoplast), finely regulates the levels of such species [4,77,96,111,116,122].

The catalase system, “the oldest known and first discovered antioxidant enzyme” [43], firstly reported in 1900 by O. Loew [47], has been proven with time to be, besides a powerful antioxidant, a main member of the global cell metabolism in all aerobic beings. For example, it has been proved that human catalase displays 245 single-nucleotide polymorphisms which are involved in diverse physiological and pathological situations [30,48,64], including hypertension, diabetes mellitus, insulin resistance, dyslipidemia, asthma, bone metabolism or vitiligo. Besides these genetic factors, CAT activity may be affected by age, physical activity, seasonal variations and certain chemical compounds [64]. Additionally, catalase was found to regulate lipid metabolism in liver without compromising the overall oxidative damage of cells [98], and the modulation of its expression in cancer cells seems to be a strategy to be potentiated for chemotherapy purposes [43].

Nevertheless, under certain physiological situations, catalase plays also a central role in processes where H_2_O_2_ and other ROS become signal molecules and need to be modulated to exert a weighted action [19,[51], [52],^59^,^113^]. Additionally, catalase is prone to undergo diverse regulation processes, from the gene expression to post-translational modifications (PTMs) which may modify the final role of the enzyme. In this work, a review of the molecular and biochemical features of catalases from different origins, mainly mammalian and plants, will be reported. An updated perspective and future prospective on the modulation of catalase by PTMs, especially those promoted by nitric oxide (NO) and derived species, and hydrogen sulfide (H_2_S) will be also provided.

## 2 Plant catalases: cell localization, structure, genes, isozymes

In eukaryotes catalase localizes in the soluble fraction of peroxisomes, namely peroxisomal matrix following the terminology used for the mitochondrial internal soluble space. Because of the prominent role played by catalase in the H_2_O_2_ metabolism, peroxisomes are considered as the main cell locus for this ROS and as eminently oxidative organelles [27,82,110,118]. This tight link between catalase and peroxisomes, supported by the composition of the polypeptide C-terminus sequence and the diverse number of methods used to localize this enzyme within the cell, has led the scientific community to consider catalase as the typical marker enzyme for these organelles.

A wide view of the hundreds of catalase gene and protein sequences that have been reported [21,49] reveals that this enzymatic system displays some diversity of structures and molecular properties. Although with some differences in the number and identity of domains, from a structural point of view catalases share a general tertiary (monomer) structure. Thus, according to the literature, native catalase is basically a tetramer with four identical subunits, whose molecular mass ranges from 220-350 kDa. The subunit size is quite similar among species ranging from 55-59 kDa and each one contains a heme group in the catalytic center [8,24,31,53,82,83,93,121]). Some exceptions to this size homogeneity have been reported in plants. Very recently, the quaternary structure of catalase from pepper (*Capsicum annuum* L.) fruits was characterized by non-denaturing electrophoresis and gel filtration chromatography. The enzyme was proved to be a homodimer of 125–135 kDa (depending on the method followed) composed by two subunits of 55 kDa each [107]. On the other hand, catalase isolated from leaves of the halophyte *Mesembryanthemun crystallinum* contained an enzyme with a molecular mass of about 320 kDa, and it was formed by two kinetically active dimeric forms of 160 kDa [89]. It must be noted that the reason for the above discrepancy among monomer sizes of those of the native proteins resides, besides the plant origin, in the experimental methods used to estimate them. These approaches include basically denaturing (monomer size) and native electrophoreses and molecular exclusion (gel filtration) chromatography.

Mammalian and plant catalases have also some important molecular differences. Thus, for example, bovine catalase contains one tightly bound NADPH group per subunit and this seems to protect the enzyme from oxidation by its substrate H_2_O_2_, thereby keeping the catalase-bound NADP fully reduced and maintaining the enzyme in an active state [61,62]. On the contrary, this feature has not been described yet for plant catalases. Some other atypical catalases having an extra flavodoxin-like C-terminal domain or manganese in the active site have been reported (for a review on this see Ref. [129]. Regarding to the genome, whereas mammalian catalase is encoded by a single gene, in higher plants catalase is encoded by a multigene family which potentially gives rise to a diversity of heterotetrameric isozymes whose number varies depending on the species [[4], [41], [56]]. Consequently, the number and expression of different CAT isozymes change during plant development, target tissue/organ and under different environmental conditions.

In the model plant *Arabidopsis thaliana* three catalase genes have been reported (*CAT-1*, *CAT-2* and *CAT*-*3*) [32,40,56,127]. *CAT1* is mainly expressed in pollen and seeds, *CAT2* in photosynthetic tissues but also in roots and seeds, while *CAT3* is associated with vascular tissues but also with senescent leaves [41,81,82,118]. Besides, *CAT2* and *CAT3* display opposite circadian profiles, while *CAT1* scarcely varied under the circadian clock. Likewise, the regulation of these catalase forms in *A. thaliana* also takes place at protein level, so diverse transcription factors modulate the activity of the enzyme [71,72,118,131,132]. The analysis of the genome in barley (*Hordeum vulgare*) [115] and peach (*Prunus persica*) [8] showed that both species contained two catalase genes. Likewise, three classes of catalase structural genes were identified by gene analysis in maize (*Zea mays*; *Cat1*, *Cat2* and *Cat3*) and rice (*Oryza sativa*; *OsCatA*, *OsCatB*, and *OsCatC*), and their expression was regulated depending on their distribution and the environmental conditions [56,103,104,108,126]. In cotton (*Gossypium* ssp.), two genes were initially reported to encode the two subunits of catalase enzymes giving a total of the five isoforms [86,87]. However, lately, the genome-wide characterization of *Gossipium hirsutum* has found that the *CAT* gene family is composed of, at least, seven genes [124]. In hot pepper (*Capsicum annuum* L.) plants three different catalase cDNA clones (*CaCat1*, *CaCat2*, and *CaCat3*) have also been identified, where *CaCat1* and *CaCat2* were regulated differently by the circadian rhythm and had different spatial distributions in leaf and stem. However, during fruit development after pollination in hot pepper, the major transcript was that of *CaCat1* [70]. Interestingly, by RNA-seq and further confirmation by iTRAQ proteomic analysis in sweet pepper fruits (*C. annuum*) at different ripening stages, three independent catalase genes have been detected [46]. Those genes corresponded to sequences previously reported in leaves, stems, roots, ovary, and fruits from hot pepper plants [67,101].

Besides playing a relevant function in plant growth, development and stress conditions [4,82,88,93], the catalase enzymatic system has been also associated to fruit ripening and postharvest events [20,55,74,90,94,95]. The three catalase proteins detected in sweet pepper fruits were composed of 492 amino acids and showed among them identities ranging 78–86%. Besides, all three isoforms contain a canonical SRL tripeptide, a characteristic peroxisomal targeting signal type 1 (PTS1), but located 9 amino acids upstream of the C-terminus (Supplementary Fig. 1; González-Gordo et al., unpublished results). The presence of an internal PTS1 has been also shown in other plant species such as pumpkin (*Cucurbita* sp.) cotyledons which comprise three catalases, the Cat1 having a functional QKL, located at −13 to −11 from the C-terminus [57].

Using cell biology and biochemical techniques for the analysis of catalase activity in plant species, basically a combination of organelle isolation, PAGE/isoelectrofocusing and western blotting approaches, a variable number of isozymes have been reported for this antioxidant enzymatic system. The enzymatic expression of the multiple isoforms of plant catalases have been reported to vary depending on the plant developmental stages and the environmental conditions [18,82]. Thus, the isoenzyme profiles described so far for plant catalase ranges from one unique isozyme, as detected in lentil (*Lens culinaris*) leaves [109] and other species, up to eight isozymes, as found in sunflower (*Helianthus annuus*) cotyledons during the transition from glyoxysomes to leaf peroxisomes [86,87]. In Table 1, the number of catalase isozymes detected in a series of plant species is given. Interestingly, although in pepper fruits three catalase cDNA clones and three protein sequences were found, only one isoenzyme band could be detected by isoelectric focusing, which displayed a variable mobility depending on its oxidation status [107].

**Table 1:** Number of catalase isoenzymes from different plant species detected through biochemical and cell biology approaches.

Plant species Can this column be widened for a better proportion of columns?	Tissue/organ/physiological/developmental conditions	Number of isozymes	References
Lentil (*Lens culinaris*)	Leaves	1	[115]
Pepper (*Capsicum annuum*)	Fruits	1	[107]
Olive (*Olea europea*)	Fruits	1	[75]
*Ziziphus mauritania* (common names either Chinese date, jujube, or Indian plum)	Fruits	2	[66]
Loblolly pine (*Pinus taeda*)	Megagametophytes	4	[83]
Pea (*Pisum sativum*)	Leaf peroxisomes	5	[24]
Cotton (*Gossypium hirsutum*)	Seeds	5	[86,87]
*Arabidopsis thaliana*	Leaves, flowers, roots	6	[32,40,78]
Sunflower (*Helianthus annuus*)	Cotyledons	8	[34]

## 3 Modulation of plant catalases by RNS and H_2_S. Post-translational modifications

Although catalase might be considered as a constitutive enzyme since it is the necessary first defense mechanism to modulate the H_2_O_2_ levels in aerobic organisms, it is also accepted as an inducible/repressible system under unfavorable conditions like those triggered under oxidative stress situations. As mentioned above, this variability of the plant catalase system is somehow linked to the differential expression patterns of each gene class, which seems to be driven by the plant developmental stage, the targeted tissue/organ and the environmental conditions [4,81,82]. In these regulating events, besides factors such as transcription factors, alternative splicing and miRNA [124], likely some sort of post-translational modification (PTM) may also participate boosting or lowering the enzyme activity depending of a plethora de factors like species, stress agent, growth and developmental stage, nutritional conditions, etc. Recently [73,133], reported the potential PTMs which fine-tunes the activity of heme proteins, including cross-link with heme side chains or porphyrin rings, amino-acid cross-links between two or more residues, heme modifications, and also alterations of amino acid by glycation, phosphorylation, acylation, oxidation/carbonylation and nitration.

Phosphorylation is a well-studied mechanism involved in many regulatory and complex processes in eukaryotes. Thus, catalase phosphorylation has been associated to regulatory situations in a number of cases in humans [17,102,134]. On the other hand, it was found that the carbonylation level of catalase and other proteins is of great importance in hypertensive obese Kolesty rats [80]. In plants, for example, the regulation of catalase by phosphorylation has been reported in *A. thaliana* under drought stress conditions [135]. In fact, phosphorylation of catalase and other proteins seems to confer resistance to plants against abiotic stress and blast disease in rice [15]. Acylation of catalase and glutathione S-transferase from rice leaves has been described to have a regulatory function in oxidative stress situations [133]. As indicated above, catalase from pepper fruits undergoes an oxidation process – a typical PTM linked to oxidative stress - during ripening and this leads to a decrease of the catalase activity and enhanced lipid peroxidation and protein oxidation in ripe fruits [20,107]. This modification by oxidation usually implies shifts in the electrophoretic mobility of proteins and carbonylation of residues associated to alterations of the protein kinetic and molecular properties [3,99]. In plant species, some other pro-oxidant situations modifying the catalase enzyme activity, either by depressing or by stimulating it, have been reported [23,92,97,105].

Lately, other PTMs promoted by reactive nitrogen species (RNS) have gained attention, basically for the intricate and complex mechanism in which they are involved. RNS are derived from nitric oxide (NO), a radical gas with important signaling significance. Three main PTMs are generated by RNS, S-nitrosation, tyrosine nitration (Tyr-NO_2_) and metal nitrosylation, being the first two processes the most studied. S-nitrosation has been also designated S-nitrosylation although the more appropriate name is S-nitrosation [51,125]. This is a reversible PTM which occurs by the binding of NO to some Cys residues of proteins and may alter the activity of enzymes, either inactivating or fostering their activity [54,125]. Nitration is a chemical reaction that enables a nitro group (-NO_2_) to bind to certain amino acid residues being tyrosine the most studied [2,9,16,20,25]. Nitration seems to be mainly mediated by the action of peroxinitrite (ONOO^−^), a strong oxidizing molecule that is formed by the reaction of NO and O_2_^·-^ [35]. An increase in the content of this PTM has been commonly considered as a nitro-oxidative stress marker and usually inhibits of the enzyme activity [26,117]. Through several experimental approaches using either affinity chromatography of vinyl sulfone silica with GSNO or immunological/proteomic analyses have also revealed that catalase from different plant species could be target of S-nitrosation and/or nitration [11,20] and also from animal cells [123]. Moreover, previous data showed that the inhibitory effect of peroxynitrite on catalase activity seems to be a common consequence independently of the origin of the catalase since, besides the above references, it has been also described in farer species as *Aspergillus niger* [63] and fish liver [114], or pepper fruits [20]. On the other hand, *in vitro* assay of plant catalases seems to indicate also that S-nitrosation negatively modulates the activity. Likewise, *in vitro* assay of tobacco and pea samples in presence of different NO-donors such as nitrosoglutathione (GSNO) or DETA NONOate showed the down-regulation of the catalase activity [22,91].

Recent data obtained in sweet pepper fruits demonstrated that catalase was prone to undergo the two RNS-derived PTMs, S-nitrosation and nitration. Both processes occur during fruit ripening by virtue of the NO and RNS dynamics and give rise to a lower enzyme activity throughout this physiological process [20,107]. The abundance study of the nitrated proteins showed catalase as the most prominent protein undergoing nitration in this plant organ [20]. This is of great importance at subcellular level and provides to peroxisomes a remarkable role in fruit ripening taking into account that these organelles in plants contain the β-oxidation pathway which generates H_2_O_2_ through the action of the first enzyme of this route, the acyl-CoA oxidase [42,107].

Unlike the above PTMs, proteins persulfidation has been less studied in plant systems, although the available data point towards an important role of this process in a number of cellular functions and metabolic pathways [37,38]. Persulfidation is promoted by hydrogen sulfide (H_2_S) which reacts with thiol groups as NO does in modifications through S-nitrosation [28]. Due to the scarce information in plant cells, data from animal systems are being used to address the research and to evaluate similar events in plants. Thus, by using proteomic analysis a series of proteins which are common to animal and plant systems have been reported to undergo persulfidation. These include actin, β-tubulin, glyceraldehyde 3-phosphate dehydrogenase (GAPDH), ATP synthase, leucine aminopeptidase and catalase [28,29]. Regarding the specific effect of this PTM on the protein functions, it was described that GADPHs Cys 50 persulfidation in mouse liver promotes an increase in its activity [84]. Likewise, GAPDH persulfidation in *Arabidopsis* leaves also enhances the enzyme activity [5]. Additionally, it was proposed that protein persulfidation may function as a protection mechanism against over-oxidation of protein thiols (RSH) through sulfenic acid (RSOH) and sulfinic acid (RSO_2_H) to sulfonic acid (RSO_3_H) [36]. Interestingly, a comparative analysis recently reported has identified a high number of proteins which are targets of both persulfidation and *S*-nitrosation in *A. thaliana* leaves, and the authors proposed a prevalence of the persulfidation events over S-nitrosation [7]. The mechanism of both PTMs involves cysteine residues and, accordingly both may exert prominent regulation roles in antioxidant enzymes. Thus, ascorbate peroxidase from diverse plant species has been described to be both S-nitrosated and persulfidated at the Cys32 position, its activity being positively affected by H_2_S [5,12,128]. Similarly, catalase from pea (*Pisum sativum*) and *A. thaliana* has also been identified as a target of *S*-nitrosation and persulfidation [5,11]. More recently, *in vitro* assays using *Arabidopsis* peroxisomes found that both H_2_S and NO inhibited catalase activity, thus participating in the regulation of H_2_O_2_ in these plant cells [29].

## 4 S-nitrosation of catalase

**Fig 1:**
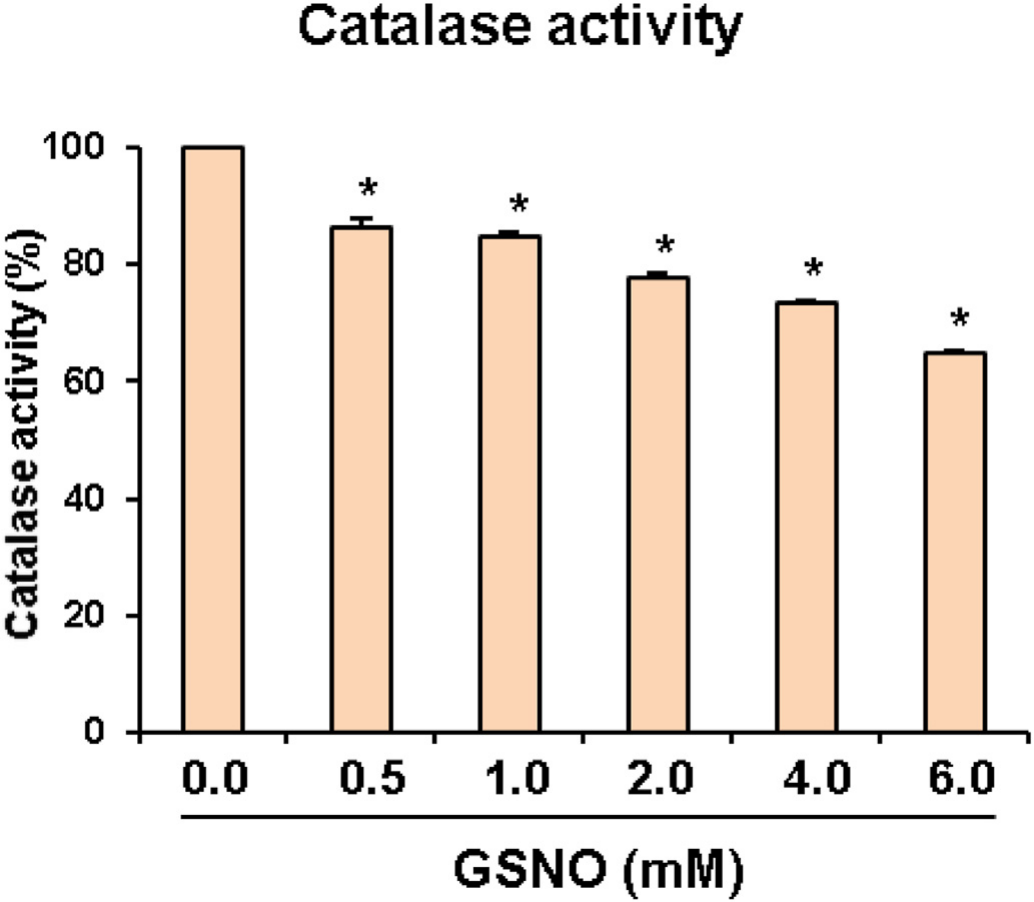
**Analysis of *Arabidopsis thaliana* catalase activity in the presence of different concentrations of S-nitrosoglutathione (GSNO)**. Aliquots of *Arabidopsis* leaf samples were pre-incubated before activity assay at 25 °C for 45 min in the presence of 2 mM GSNO (nitric oxide donor). The catalase specific activity of the untreated samples was 50.1 μmol H_2_O_2_ · min^−1^ · mg^−1^ protein. The remaining catalase activity (expressed as %) after the treatment was plotted. Catalase activity was determined according to Ref. [1]. Results are the mean of at least three biological replicates (with triplicate assays each) ± SEM. ∗Differences in relation to control values were significant at *P < 0.05*.

Regarding S-nitrosation, free NO can bind directly to the thiol group of cysteine and generate a family of molecules designated as S-nitrosothiols. One of the most relevant S-nitrosothiols comes from the interaction of NO with reduced glutathione to form S-nitrosoglutathione (GSNO). This molecule can release NO which makes it a suitable donor for the same S-nitrosation purposes, conferring GSNO a NO reservoir role [6,13,14,25]. In a study carried out in leaf samples from the plant model *Arabidopsis thaliana*, the cysteine residues which were potentially more susceptible to be S-nitrosated were identified. GSNO was used as NO donor for such studies and further computational analysis was applied. Previously, it was found that GSNO negatively affected the activity of catalase activity of *A. thaliana* leaves by reducing it up to 25–30% in a concentration-dependent manner (Fig. 1).

**Fig. 2:**
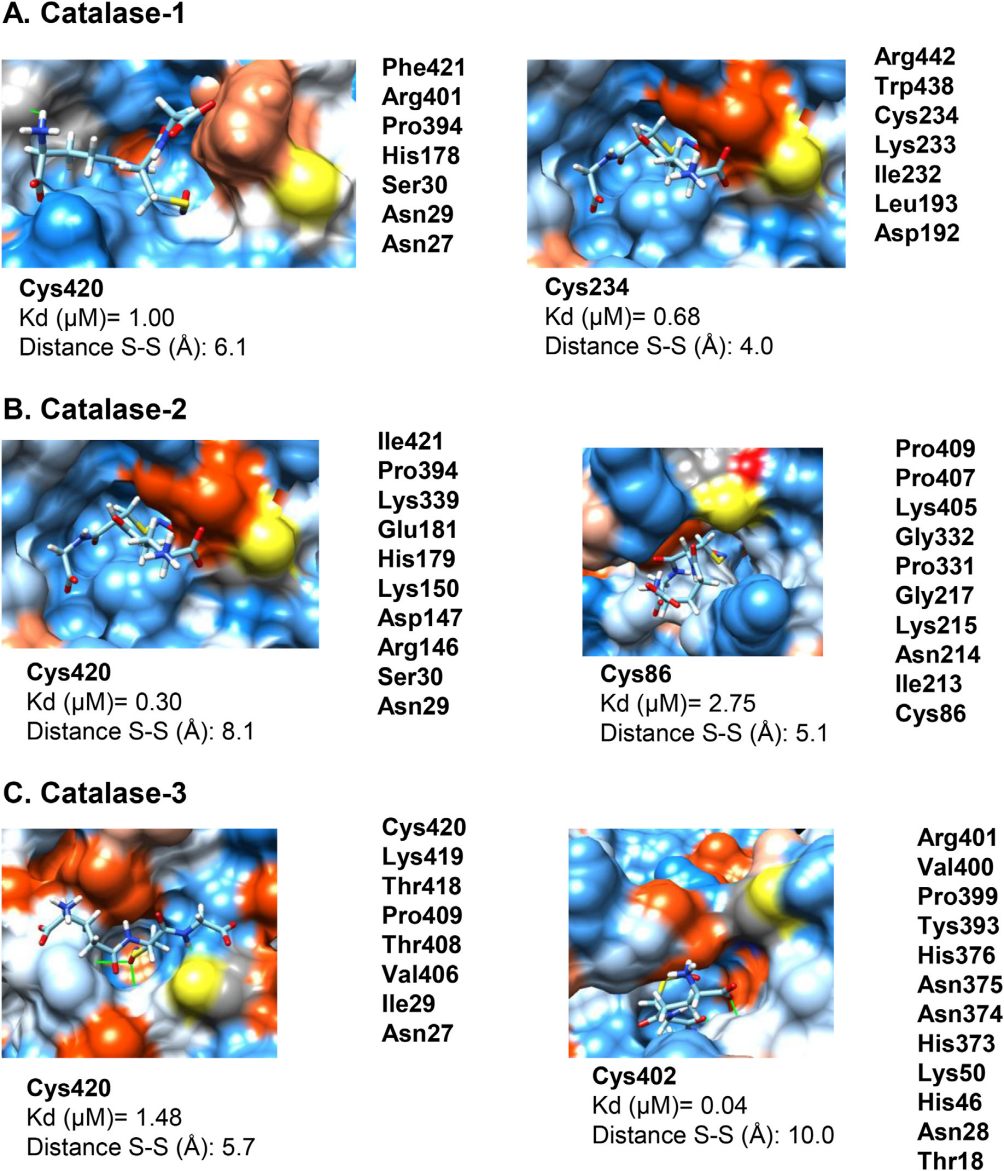
**Blind docking of GSNO to *Arabidopsis thaliana* catalase**. Poses of the docking of GSNO on catalase isozymes from *A. thaliana* near to Cys (sulfur atom in yellow), showing the predicted Kd calculated from the estimated ΔG of the interaction and the distance with the sulfur atom from GSNO. The surface of the protein is colored as a function of the Kyte-Doolittle scale. Colors range from dodger blue to white for the most hydrophilic and to orange-red for the most hydrophobic. Residues involved in the interaction with GSNO are listed on the right side of each image.

To gain deeper insight into the mechanism of modulation of this activity by NO, computational study was carried out by docking GSNO to the quaternary structure of Arabidopsis CAT-1, CAT-2 and CAT-3 proteins and analyzed by homology modeling [76; early display in the XI Meeting of the Spanish Group for Free Radical Research, GEIRLI]. The three *A. thaliana* catalase isozymes consist of 492 residues with a high degree of identity. CAT-1 and CAT-2 share an identity of 86.8% and 6 Cys residues, whereas CAT-3, with 7 Cys residues shares 78–79% identity with the other isozymes. The alignment of the primary structures of the isozymes revealed that the position of the different Cys is not fully preserved ([33], Supplementary Fig. 2). Then, *in silico* studies were carried out (experimental details in Supplementary Materials and methods 1). The quaternary structure of the *Arabidopsis* catalases was computed and blind docking (i.e. over the entire surface of the protein, without predetermining any preferential region) of GSNO with the models of three catalases yielded different poses ranked on the basis of the full fitness parameter [50]. When the distance between the S atoms of GSNO and Cys was introduced as a discriminant criterion, the solutions were reduced to two poses with different estimated affinity for each isoenzyme, being Cys420 a putative S-nitrosation target common to all of them (Fig. 2). The site close to Cys420 was named S1 and the others S2. From the computed values of ΔG the Kd of the interaction of GSNO with catalases was estimated. It is important to emphasize that although the comparison of the estimated Kd for a ligand with different molecules is not straightforward, the fact that the three catalases share Cys420 as target makes possible the evaluation of the relative affinity of sites 1 and 2 (i.e. S2/S1 ratio), being 0.68, 9.17 and 0.03 for CAT-1, CAT-2 and CAT-3, respectively. The outstanding value for CAT-3 may be biased by the fact that the position of the S atoms is at the upper limit of the tolerance to be considered as feasible. The relative affinity values suggest that the affinity of GSNO is about one-fold higher for site 1 of CAT-2 and slightly higher for site 2 in CAT-1. Interestingly, CAT-1 has been reported as an important player in the removal of H_2_O_2_ generated under various environmental stresses and the role of the glutathione status in transmitting signals derived from intracellular H_2_O_2_ was postulated [76].

More recently, commercial catalase from bovine liver (527 aminoacids) was used as model protein to investigate the S-nitration events by the biotin-switch labeling approach, in-gel trypsin digestion, and nanoliquid chromatography coupled to mass spectrometry. It was found that, upon incubation with S-nitroso-l-cysteine, the bovine enzyme could potentially undergo S-nitrosation at the Cys377 in the peptide sequence L_366_GPNYLQIPVNC_377_PYR_380_ [107]. The length and putative S-nitration predicted sites for *A. thaliana* catalase clearly differ from the bovine protein. However, the three *A. thaliana* catalases contain a 15-aminoacid peptide which shares 11 amino acids with the one from the bovine fragment with a cysteine located at position 370. Likewise, catalase from pepper fruits also contain a similar peptide, hence a potential S-nitration at Cys_370_ was postulated [107]. On the other hand, in the Arabidopsis docking assays, GSNO was used as the NO donor whereas in the bovine catalase was nitrosated using Cys-NO as agent, but both GSNO and Cys-NO have different reactivity and recognition features. More research in this subject should be necessary.

**Fig. 3:**
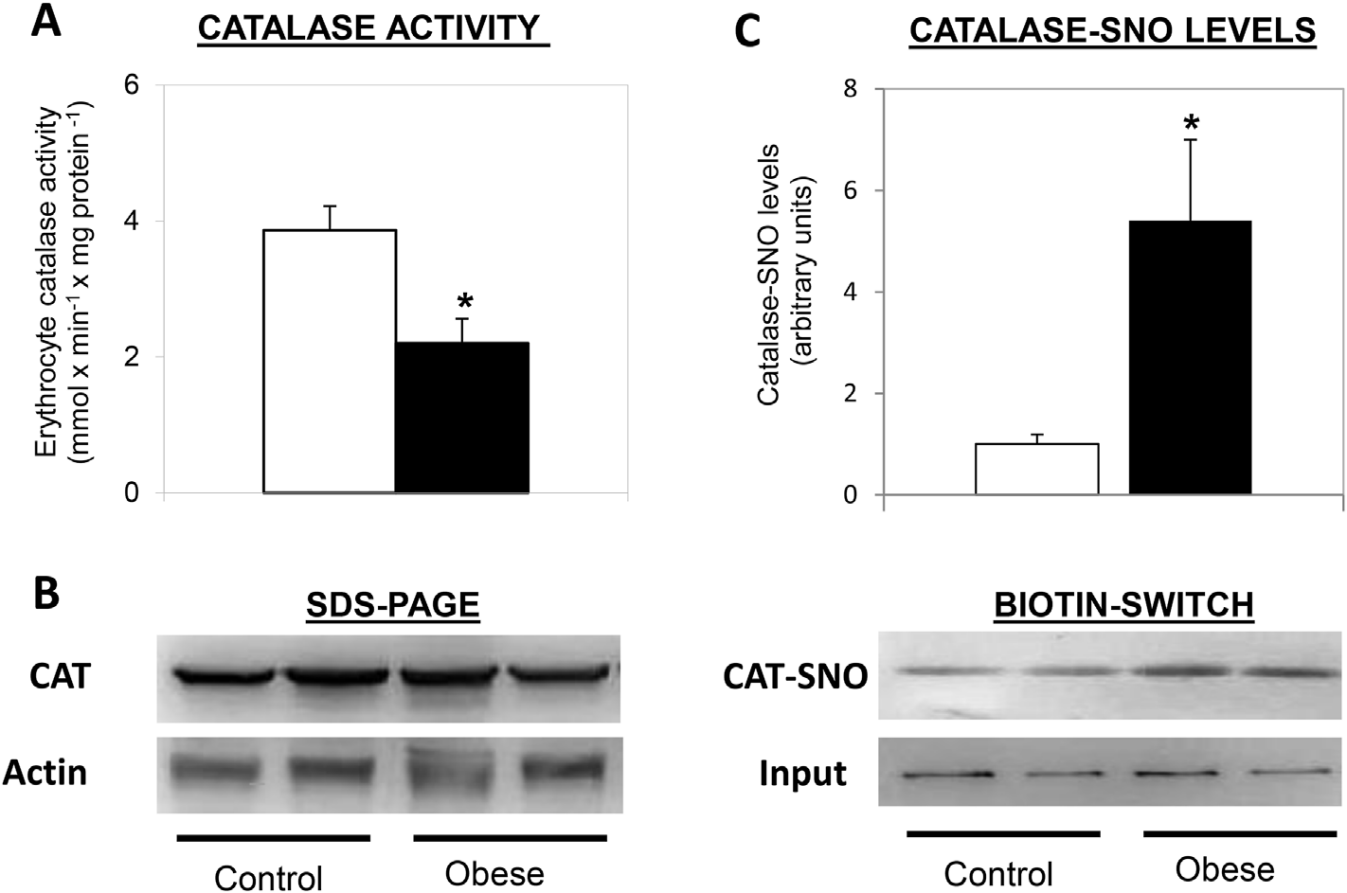
**Erythrocyte catalase in healthy and obese children**. The enzyme activity (A), protein expression (B) and nitrosation modification (C) were analyzed after an overnight fast in red blood cells of healthy (white bars) and obese (black bars) children. Catalase activity was determined according to Ref. [1]. Values represent means ± SEM. *P < 0.05. SDS was performed in 12% acrylamide gels. Antibodies used were Anti-CAT XP® (Cell Signaling) and Anti-β-actin (Abcam). For detection of nitrosated catalase (catalase-SNO), the biotin-switch method for nitrosthiols groups (SNO) was followed. Data from biotin-switch were quantified by the ImageJ programme.

Unique cases of catalase S-nitration have been also found in erythrocytes of obese children. Erythrocytes have recently been proposed as systemic oxidative stress sensors in humans. They show increased levels of oxidative stress damage in many common physio-pathological situations such as obesity, insulin resistance and chronic inflammation. Such changes in oxidative stress markers appear intimately linked to a depletion in the antioxidant capacity of the erythrocytes, in which catalase inactivation plays a central role [68]. Since these blood cells lack mitochondria, the continuous production of ROS and RNS observed within them come from the high partial pressure of oxygen present in arterial blood, and the abundance of ferrous iron present as part of haemogloblin or catalase heme group [10]. Among the erythrocyte peroxidases, catalase plays a predominant role in situations in which either endogenous or exogenous hydrogen peroxide concentration exceed 10^−6^ mol/L [79]. This seems to be intimately related to redox equivalents availability (NADH and/or NADPH) within the erythrocyte [65,130]. In childhood obesity, the erythrocytes suffer chronic subclinical oxidative stress, characterized by a generalized antioxidant depletion. Catalase activity is inhibited in this condition, rendering an inefficient activation after acute sugar ingestion, particularly evident in insulin resistant children [68]. Since mature erythrocytes lack nuclei and are, thus, unable to synthesize proteins, PTMs may plausibly play a key role in this CAT activity down-regulation. Results from our group suggest that S-nitrosation may be at least one of such catalase modifications inducing this decreased activity (Fig. 3, [45; early display in the 42nd Congress of the Spanish Society of Biochemistry and Molecular Biology]). Thus, although the catalase protein level is the same in obese children and controls (Fig. 3B), the enzyme activity is reduced in the obese (Fig. 3A). Conversely, the use of biotin-switch approach to detect nitrosothiol groups bound to catalase, showed that the S-nitrosated (SNO)-catalase was higher in obese with respect to control individuals (Fig. 3C).

## 5 Conclusions

Catalase is one of the oldest and best known enzymes and its genetic, molecular and biochemical features from a large diversity of organisms have been broadly reported. The research where catalase has been investigated covers various science categories such as Biochemistry and Molecular Biology, Medicine, Pharmacology, Toxicology, Cell Biology and Food Science Technology, among many others. This means that catalase is still the main focus of many research groups as the number of references per year depicts an exponential curve since early XX century. Regarding plant biology, the enzyme is involved in many processes including seed germination, plant growth and development, senescence, fruit ripening, response to both biotic and abiotic factors, and others. In spite of this consistent background, further research is necessary to better understand some aspects related to modulation of catalase from the gene expression to the final role within cells. Regarding this last issue, accumulating evidence indicate that catalase enzyme activity is regulated via PTMs such as phosphorylation, glycation, acetylation, oxidation, and more recently by signaling events promoted by NO and derived RNS and H_2_S. The precise mechanism of regulation by NO and H_2_S remains to be elucidated. In fact, some competition of the two signaling molecules for the same cysteine targets takes place, and this makes this subject more complex to be analyzed although exciting. This has to be viewed from the perspective of the prevailing developmental and environmental conditions which may condition the endogenous generation rates of both chemicals. Besides, the presence of catalase within peroxisomes, a nitro-oxidative organelle where, besides NO and H_2_S [27,29], other molecules are also formed, assigns to this cell compartment a central signaling role in cell physiology. Finally, a deep knowledge on how catalase carries out its function within the cell and how it can be modified by certain conditions is essential to understand and to later tap into its potential biomedical and biotechnological purposes.

## Declaration of competing interest

The authors declare that the research was conducted in the absence of any commercial or financial relationships that could be construed as a potential conflict of interest.
