# Supplementary material for: Plant catalases as NO and H2S targets
Source: Redox Biol. 2020 May 25;34:101525. doi: 10.1016/j.redox.2020.101525 (PMC7276441; doi:10.1016/j.redox.2020.101525)
Supplement: Multimedia component 1 [file mmc1.docx]

**Supplementary Materials and methods 1**

**In silico Assays**

The modeling of the quaternary structure of the *Arabidopsis* catalases was approached by homology modeling at different servers (McGuffin et al., 2015; Yang et al., 2015; Fernandez-Fuentes et al., 2007; Källberg et al., 2012; Waterhouse et al., 2018), and the quality of the models was analyzed at SAVES server (https://servicesn.mbi.ucla.edu/SAVES/) in terms of non-bonded interactions by Errat (Colovos & Yeates, 1993), model compatibility with the amino acid sequence by Verify_3D (Bowie et al., 1991; Luthy et al., 1992), Ramachandran Plot (Ramachandran et al., 1963) and s Q-mean (Benkert et al., 2008) and Z-score (Benkert et al., 2011) computed by QMEAN Server for Model Quality Estimation (Benkert et al., 2009). Models output by Swiss-model showed the best overall score. The PDB structure used as template was 4qol (subunit A), that shares 52.88%, 53.59% and 58.53% identity with CAT1, CAT2 and CAT3, respectively, the coverage being 0.95 for CAT1 and CAT3 and 0.96 for CAT2. Docking experiments were carried out at SwissDock (Grosdidier et al., 2011) using the quaternary structure of the best model and the coordinates of GSNO deposited at ZINC database (Irwin & Shoichet, 2005), using the entire surface of the protein without predetermining any preferential region (i.e. blind docking). Results were analyzed with the help of UCSF Chimera 1.10.1 (Pettersen et al., 2004) and Kd were computed from the estimation of ΔG output by the docking algorithm.

**References**

Benkert P, Tosatto SCE, Schomburg D (2008) QMEAN: A comprehensive scoring function for model quality assessment. Prot Struct Funct Bioinf 71: 261-277.

Benkert P, Künzli M, Schwede T (2009) QMEAN Server for protein model quality estimation. Nucleic Acids Res 37: W510-W514.

Benkert P, Biasini M, Schwede T (2011) Toward the estimation of the absolute quality of individual protein structure models. Bioinformatics 27: 343-350.

Bowie JU, Luthy R, Eisenberg D (1991) A method to identify protein sequences that fold into a known three-dimensional structure. Science 253: 164-170.

Colovos C, Yeates TO (1993) Verification of protein structures patterns of nonbonded atomic interactions. Protein Sci 2: 1511-1519.

[Fernandez-Fuentes N](https://www.ncbi.nlm.nih.gov/pubmed/?term=Fernandez-Fuentes%20N%5BAuthor%5D&cauthor=true&cauthor_uid=17517764), [Madrid-Aliste CJ](https://www.ncbi.nlm.nih.gov/pubmed/?term=Madrid-Aliste%20CJ%5BAuthor%5D&cauthor=true&cauthor_uid=17517764), [Rai BK](https://www.ncbi.nlm.nih.gov/pubmed/?term=Rai%20BK%5BAuthor%5D&cauthor=true&cauthor_uid=17517764), [Fajardo JE](https://www.ncbi.nlm.nih.gov/pubmed/?term=Fajardo%20JE%5BAuthor%5D&cauthor=true&cauthor_uid=17517764), [Fiser A (2007)](https://www.ncbi.nlm.nih.gov/pubmed/?term=Fiser%20A%5BAuthor%5D&cauthor=true&cauthor_uid=17517764) M4T: a comparative protein structure modeling server. [Nucleic Acids Res](https://www.ncbi.nlm.nih.gov/pubmed?Db=pubmed&Cmd=ShowDetailView&TermToSearch=17517764&ordinalpos=1&itool=EntrezSystem2.PEntrez.Pubmed.Pubmed_ResultsPanel.Pubmed_RVDocSum)  5: W363-W368.

Grosdidier A, Zoete V, Michielin O (2011) SwissDock, a protein-small molecule docking web service based on EADock DSS. Nucleic Acids Res 39: W270-W277.

Irwin JJ, Shoichet BK (2005) ZINC-a free database of commercially available compounds for virtual screening. J Chem Inf Model 45: 177-182.

Källberg M, Wang H, Wang S, Peng J, Wang Z, Lu H, Xu J (2012) Template-based protein structure modeling using the RaptorX web server. Nature Protocols 7: 1511-1522.

Luthy R, Bowie JU, Eisenberg D (1992) Assessment of protein models with three-dimensional profiles. Nature 356: 83-85.

McGuffin LJ, Atkins J, Salehe BR, Shuid AN, Roche DB (2015) IntFOLD: an integrated server for modelling protein structures and functions from amino acid sequences. Nucleic Acids Res 43: W169-W173.

Pettersen EF, Goddard TD, Huang CC, Couch GS, Greenblatt DM, Meng EC, Ferrin TE (2004) UCSF Chimera - a visualization system for exploratory research and analysis. J Comput Chem 25: 1605-1612.

Ramachandran GN, Ramakrishnan C, Sasisekharan V (1963) Stereochemistry of polypeptide chain configurations. J Mol Biol 7: 95-99.

Waterhouse A, Bertoni M, Bienert S, Studer G, Tauriello G, Gumienny R, Heer FT, de Beer TAP, Rempfer C, Bordoli L, Lepore R, Schwede T (2018) SWISS-MODEL: homology modelling of protein structures and complexes. Nucleic Acids Res 46: W296-W303.

Yang J, Yan R, Roy A, Xu D, Poisson J, Zhang Y (2015). The I-TASSER Suite: Protein structure and function prediction. Nature Methods 12: 7-8, 2015.
