# Supplementary material for: Plant catalases as NO and H2S targets
Source: Redox Biol. 2020 May 25;34:101525. doi: 10.1016/j.redox.2020.101525 (PMC7276441; doi:10.1016/j.redox.2020.101525)
Supplement: Multimedia component 2 [file mmc2.pdf]

```
CaCat1      MDLSKYRPSSAYDSPFLTTNAGGPVYNNVSSLTVGPRGPVLLEDYHLIEKLATFVRERIP      60
CaCat3      MDPYKHRPSSAANSPYWTTNSGAPVYNNNSSLTVGARGPILLEDYHLLEKLANFDRERIP      60
CaCat2      MDPYKYRPSSAFNSPFCTTNSGAPVWNNNSSLTVGARGPVLLEDYHLVEKLANFDRERIA      60
            **  *:***** :**: ***:*.**:** ****** ***:*******:****.* ****

CaCat1      ERVVHARGASAKGFFEVTHDISHLTCADFLRAPGVQTPVICRFSTVVHERGSPESIRDIR      120
CaCat3      ERVVHARGASAKGFFEVTHDITRLTCADFLRAPGVQTPVIVRFSTVIHERGSPETLRDPR      120
CaCat2      ERVVHARGASAKGFFEVTHDITHLTCADFLRAPGVQTPVIVRFSTVIHERGSPETLRDPR      120
            *********************::***************** *****:*******::** *

CaCat1      GFAVKFYTREGNFDLVGNNVPVFFNRDAKSFPDTIRALKPNPKSHIQENWRILDFFSFLP      180
CaCat3      GFAVKFYTREGNFDLVGNNFPVFFVRDGMKFPDMVHALKPNPKSHIQENWRILDFFSHHP      180
CaCat2      GFAVKFYTREGNFDLVGNNFPVFFIRDGMKFPDMVHSLKPNPKSHIQENWRVLDFFSHHP      180
            *******************.**** **. .*** :::**************:*****. *

CaCat1      ESLHTFAFFYDDVCLPTDYRHMEGFGVHAYQLINKAGKAHYVKFHWKPTCGVKSMTEEEA      240
CaCat3      ESLHMFSFLFDDLGIPQDYRHMEGSGVNTYTLINKAGKAHYVKFHWKPTCGVKCLLDDEA      240
CaCat2      ESLHMFTFLFDDIGIPQDYRHMDGSGVHTFTLINKAGKSTYVKFHWRPTCGVKSLLEEEA      240
            **** *:*::**: :* *****:* **::: *******: ******:******.: ::**

CaCat1      IRVGGTNHSHATKDLYDSIAAGNYPEWKLFIQIMNPEDVDKFDFDPLDVTKTWPEDILPL      300
CaCat3      VKVGGTNHSHATQDLYDSIAAGNYPEWKLFIQTIDPDHEDKFDFDPLDVTKIWPEDILPL      300
CaCat2      IRVGGANHSHATQDLYDSIAAGNYPEWKLFIQTMDPAHEDRFDFDPLDVTKTWPEDIFPL      300
            ::***:******:******************* ::* . *:********** *****:**

CaCat1      MPVGRLVLNRNIDNFFAENEQLAFNPGHIVPGVYYSEDKLLQTRIFAYADTQRHRIGPNY      360
CaCat3      QPVGRLVLNKNIDNFFVENEQLAFCPAIIVPGIHYSDDKLLQTRVFSYADTQRHRLGPNY      360
CaCat2      QPVGRLVLNKNIDNFFNENEQLAFCPSIVVPGVYYSDDKMLQTRIFSYSDTQRYRLGPNY      360
             ********:****** ******* *. :***::**:**:****:*:*:****:*:****

CaCat1      MQLPVNAPKCAHHNNHRDGAMNFMHRDEEVDYLPSRFDPCRPAEQYPIPSCVLTGRREKC      420
CaCat3      LQLPPNAPKCAHHNNHHEGFMNFMHRDEEVNYFPSRFDPCRHAEQYPIPPRVLNGKRDRC      420
CaCat2      LQLPANAPKCAHHNNHYDGSMNFMHRDEEIDYFPSRYDPVRHAENYPIPTTVCSGKREKC      420
            :*** *********** :* *********::*:***:** * **:****  * .*:*::*

CaCat1      VIPKENNFKQAGERYRSWAPDRQDRYINKWVESLSDPRATHEIRSIWISYLSQADKSCGQ      480
CaCat3      IIQKENNFKQPGERYRSWAPDRQERFICRWVDALSEPRVTHEIRTIWLSYWSQADKSLGQ      480
CaCat2      IIQKENNFQQPGERYRSFSADRQERFIRRWVEALSDPRITYEIRSIWISYWSQADKSLGQ      480
            :* *****:* ******:: ***:*:* :**::**:** *:***:**:** ****** **

CaCat1      KVASRLTVKPTM          492
CaCat3      KLASRLNVKPTM          492
CaCat2      KLASRLNVRPSI          492
            *:****.*:*::
```

Supplementary Figure 1. Alignment of the three *Capsicum annuum* catalase protein sequences. The amino acid sequences of CAT1, CAT2, and CAT3 were obtained from RNA-Seq and iTRAQ data and were aligned using MUSCLE (Edgar 2004). BLAST searches were made with the National Center for Biotechnology Information Web site (http://www.ncbi.nlm.nih.gov/). The accession number for each catalase isozenzyme were Q9M5L6 for Catalase I (CaCat1), A0A1U8FMA2 for Catalase II (CaCat2) and 0A1U8GAA6 for Catalase III (CaCat3). The potential PTS1 (SRL), shared by the three sequences, is framed at aminoacids 484-487.

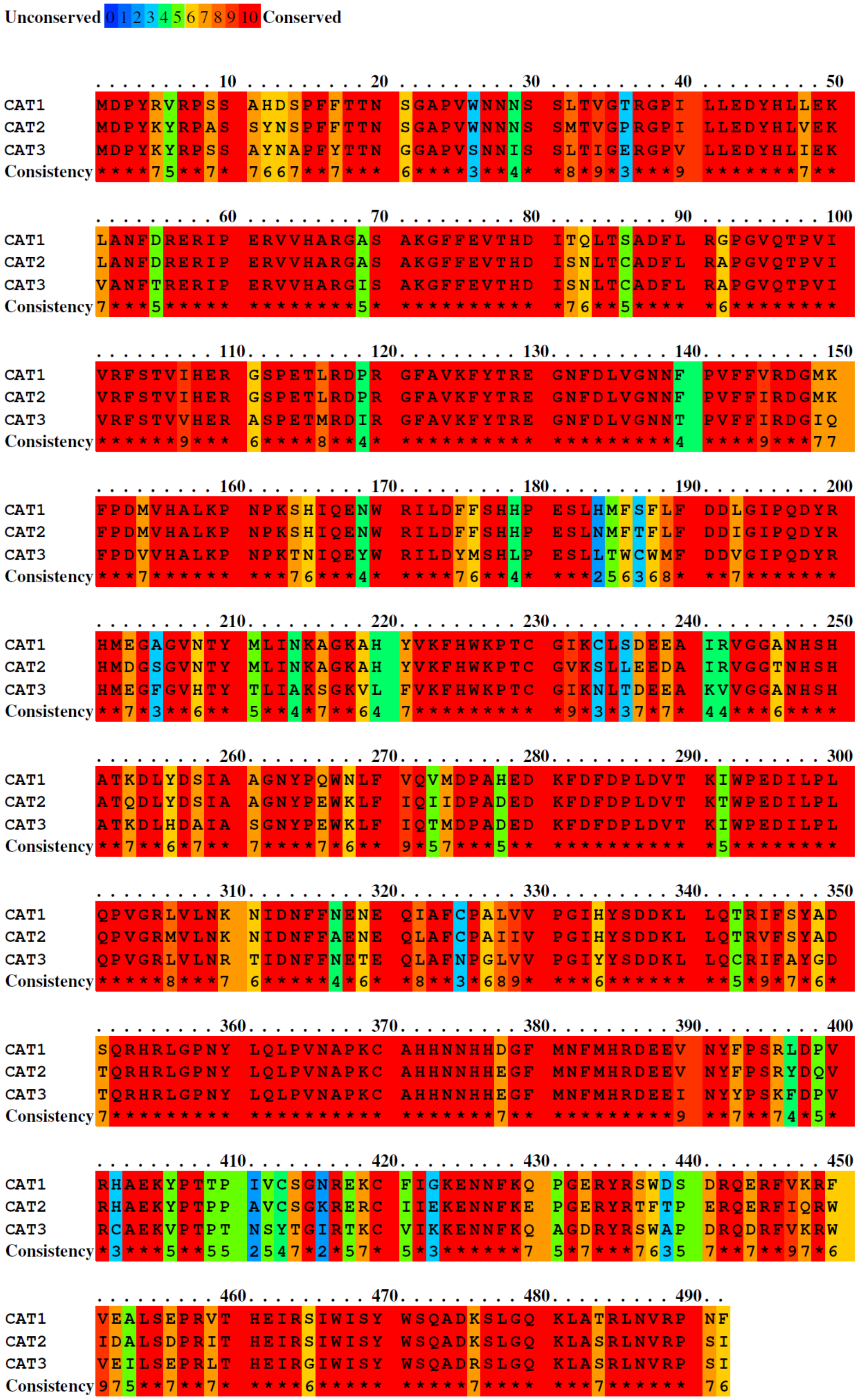

Supplementary Figure 2. Alignment of the three *Arabidopsis thaliana* catalase protein sequences. The amino acid sequences of CAT1, CAT2, and CAT3 were aligned using MUSCLE (Edgar 2004). BLAST searches were made with the National Center for Biotechnology Information Web site (http://www.ncbi.nlm.nih.gov/). The accession number for each catalase isozenzyme were AEE29998 for CAT 1, AEE86462 for CAT2 and AEE29995 for CAT3. The alignment of the primary structures reveals the high degree of identity (>75%) of the three catalases from *Arabidopsis thaliana* although the position of the different Cys is not fully preserved.
